# Dual Aetiology of Diabetes Insipidus in Pregnancy: Vasopressinase‐Mediated and Central Mechanisms

**DOI:** 10.1155/crin/7536497

**Published:** 2026-06-19

**Authors:** Philani Ezekiel Mkhize, Pauli van Heerden, Tholakele Sabela, Mogamat Razeen Davids, Mogamat-Yazied Chothia

**Affiliations:** ^1^ Department of Medicine, Division of Nephrology, Faculty of Medicine and Health Sciences, Stellenbosch University, Cape Town, South Africa, sun.ac.za; ^2^ Department of Obstetrics and Gynaecology, Faculty of Medicine and Health Sciences, Stellenbosch University, Cape Town, South Africa, sun.ac.za

**Keywords:** desmopressin, diabetes insipidus, gestation, vasopressinase

## Abstract

Diabetes insipidus (DI) is a heterogeneous disorder characterised by polyuria and polydipsia due to impaired arginine vasopressin (AVP) secretion or action. We describe a 36‐year‐old pregnant woman who presented at 28 weeks’ gestation with weakness, severe hypokalaemia from renal potassium wasting, hypernatraemia and polyuria with dilute urine. Vasopressinase‐mediated DI was suspected; there was no response to AVP, but desmopressin (dDAVP) produced a rapid clinical improvement, confirming the diagnosis. However, postpartum recurrence of symptoms prompted further evaluation, with imaging suggestive of lymphocytic infundibuloneurohypophysitis, necessitating reinitiation of dDAVP. This case highlights the diagnostic complexity of DI in pregnancy and the need to remain vigilant for central causes, even when vasopressinase‐mediated DI is initially suspected.

## 1. Introduction

Diabetes insipidus (DI) is a heterogeneous disorder characterised by polyuria and polydipsia resulting from either inadequate secretion of arginine vasopressin (AVP) or impaired renal responsiveness to it. DI during pregnancy is uncommon, with an estimated incidence of 4 per 100,000 pregnancies [1]. It may be unmasked by the physiological changes in osmoregulation that occur during pregnancy [2] or arise de novo due to placental vasopressinase (PVP), which accelerates the clearance of endogenous AVP [3, 4].

PVP is an aminopeptidase produced by trophoblasts during normal gestation and increases the metabolic degradation of AVP. Its circulating levels correlate with placental mass and gestational age [5]. Pregnancy is also characterised by plasma volume expansion [6], driven by complex osmoregulatory adaptations mediated by neurohormonal factors such as human chorionic gonadotrophin and relaxin. These are associated with systemic vasodilation and relative hypotension, leading to nonosmotic AVP release, a reduced osmotic threshold for AVP secretion and increased thirst [7–9].

Collectively, these changes establish a new osmotic setpoint, resulting in a reduction in serum osmolality and sodium concentration by approximately 10 mOsm/kg and 5 mmol/L, respectively, early in the first trimester and persisting until term [10, 11]. Concurrently, rising PVP levels help maintain fluid balance by enhancing AVP clearance. Given the heterogeneity of DI in pregnancy, identifying the underlying mechanism is essential, as it directly influences both the choice and duration of therapy.

We report the case of a 36‐year‐old multiparous woman who was referred during pregnancy with progressive muscle weakness and was subsequently found to have severe hypokalaemia and polyuria attributable to both PVP and a central cause of DI.

## 2. Case Report

A 36‐year‐old woman, gravidity 4 and parity 3, was referred at 28 weeks of dichorionic diamniotic (DCDA) gestation with a 5‐day history of progressive generalised muscle weakness and slurred speech. Her first antenatal visit at 10 weeks of gestation had revealed Class III obesity (a body mass index of 35.7 kg/m^2^ [height 146 cm, weight 76 kg]), a 5‐pack‐year smoking history and alcohol consumption of an average of 16.2 units per week. She had an unremarkable previous obstetric history and normal renal biochemical testing.

During the current admission, she denied any prodromal symptoms, exposure to drugs or toxins, pica or dietary changes, including salt cravings. Physical examination revealed a blood pressure of 116/88 mmHg and generalised muscle weakness. She had multiple healing lacerations and abrasions on her knees following fall episodes because of muscle weakness. Ultrasound revealed normal foetal growth with normal Doppler indices and amniotic fluid volumes for both twins. Following the placement of a urinary catheter, it became apparent that she had polyuria with a urine volume of 7310 mL per day.

Urine dipstick revealed no active sediment or glycosuria. Basic laboratory investigations revealed serum sodium 154 mmol/L, potassium 2.9 mmol/L, chloride 115 mmol/L, urea 1.3 mmol/L, creatinine 45 μmol/L and serum osmolality of 309 mOsm/kg of water. An arterial blood gas obtained on Day 5, while the patient was receiving potassium supplementation and the serum potassium remained low (2.9–3.2 mmol/L), showed a pH of 7.39 and a bicarbonate of 18.9 mmol/L, indicating no evidence of metabolic alkalosis. Liver enzyme profile was in keeping with alcoholic hepatitis with an aspartate aminotransferase (AST) of 248 IU/L and alanine aminotransferase (ALT) of 133 IU/L, and an AST:ALT ratio of 1.9, with an elevated gamma glutamyl transferase of 55 IU/L. Liver ultrasound revealed normal size and echogenicity. Macrocytosis (a mean cell volume of 104.7 fL), with a normal platelet count, was noted on the full blood count.

Urine chemistry demonstrated dilute urine and renal potassium wasting, with a urine osmolality of 101 mOsm/kg H_2_O and a urine potassium‐to‐creatinine ratio (uK/Cr) of 4.4 mmol/mmol; a uK/Cr > 2.0 mmol/mmol in the setting of hypokalaemia is consistent with renal potassium loss. The absence of hypertension and metabolic alkalosis (pH 7.39, HCO_3_^-^ 18.9 mmol/L), together with low renin (17.3 mIU/L; normal supine range 2.8–39.9 mIU/L) and aldosterone (27.0 pmol/L; normal supine ≤ 443 pmol/L) levels, made hyperaldosteronism or hyperaldosterone‐like states unlikely. Urine electrolytes further indicated low distal sodium delivery during hypokalaemia, with urine sodium ranging from < 20 to 32 mmol/L and urine chloride from < 20 to 33 mmol/L. The uK/Cr ratio ranged from 4.4 to 9.1, remaining persistently > 2.0 and supporting ongoing urinary potassium wasting. Intravenous potassium supplementation had been commenced 9–10 days prior to nephrology review. By Day 9, the serum potassium had normalised to 3.8–4.1 mmol/L; however, polyuria persisted, with urine output ranging from 261 to 290 mL/h, and urine osmolality remaining low at 110–215 mOsm/kg of water.

**FIGURE 1:**
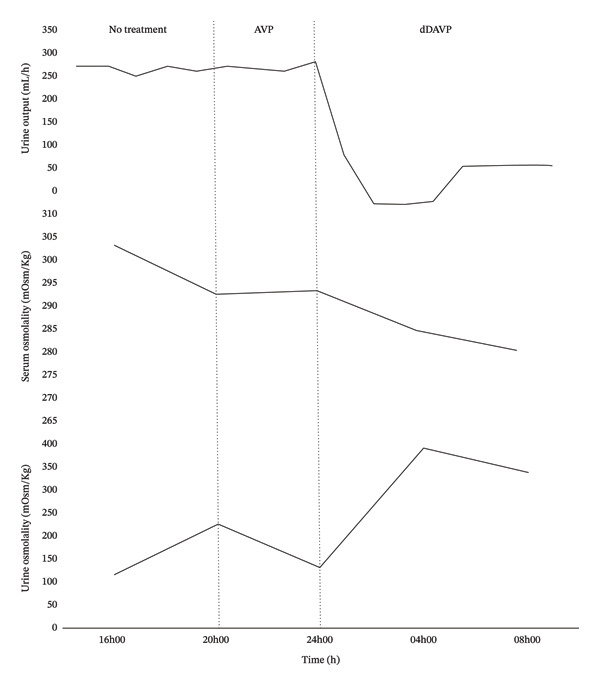
Changes in hourly urine volume (mL/hour), serum osmolality (mOsm/kg of water) and urine osmolality (mOsm/kg of water) over a period of 16 h; no intervention during the first 4 h of observation, administration of subcutaneous AVP at 20h00 and administration of subcutaneous dDAVP at 24h00. The dotted lines indicate the timing of the administration of AVP and dDAVP. AVP, arginine vasopressin; dDAVP, desmopressin.

Given the patient’s clinical presentation, there was a high index of suspicion for the presence of increased circulating PVPs. This was demonstrated following a lack of urinary concentration after the administration of 20 IU (1 mL) subcutaneous AVP but a rapid response after the administration of 2 μg (0.5 mL) subcutaneous desmopressin (dDAVP). The average hourly urine output prior to dDAVP administration was 277 mL/hr (4.6 mL/min). Following the administration of dDAVP, the average urine output decreased to 56 mL/hr (0.9 mL/min). This trend was sustained for 4 h after dDAVP administration with a subsequent gradual increase in urine volumes as the effect diminished (Figure 1). She was prescribed twice daily 2 μg of subcutaneous dDAVP and was later switched to the oral formulation.

The serum sodium concentration decreased and ranged between 131 and 140 mmol/L for the remainder of pregnancy. Although hepatic transaminitis improved, there was no need to reduce the dose of oral dDAVP. The patient underwent an uncomplicated caesarean section at 37 weeks’ gestation. The neonates were well grown, had good Apgar scores and experienced an uneventful neonatal course.

The patient was followed for 2 months during the postpartum period, during which time dDAVP was discontinued. During this interval, she reported no symptoms suggestive of DI, such as polyuria or polydipsia; however, 2 months after the last follow‐up visit, she re‐presented with severe hypokalaemia and hypernatraemia, accompanied by urine volumes of 3–4 L/day. DDAVP was subsequently restarted, resulting in improvement in the serum sodium concentration and polyuria.

Although the patient denied local symptoms suggestive of pituitary enlargement, magnetic resonance imaging of the pituitary gland was performed. Imaging demonstrated normal pituitary dimensions (9 mm [height] × 12 mm [width] × 10 mm [anteroposterior]); however, there was thickening (4 mm) and enhancement of the pituitary stalk at the level of the optic chiasm (normal < 3 mm), together with loss of the normal T1‐weighted posterior pituitary with fat suppression ‘bright spot’. Tests of anterior pituitary hormones, including adrenocorticotropic hormone, thyroid‐stimulating hormone and growth hormone, remained intact. These findings, along with the history of recent pregnancy, were highly suggestive of lymphocytic infundibuloneurohypophysitis (LINH). A pituitary biopsy was not performed given the characteristic MRI findings. Three months later, the patient remained on dDAVP and was clinically stable with resolution of symptoms.

## 3. Discussion

Our initial concern was that the DI was driven by excessive production of PVP. Pregnancy‐related risk factors that may lead to excessive PVP activity include increased placental synthesis and reduced vasopressinase hepatic clearance. In this case report, two risk factors were identified. First, increased synthesis with PVP‐mediated DI in the form of DCDA gestation. Second, reduced hepatic metabolism due to the presence of alcoholic hepatitis. It is important to note that there was no causal relationship between alcohol intake and the patient’s presentation, as her symptoms preceded her latest alcohol binge. Other causes of hepatitis that were excluded were haemolysis, elevated liver enzymes and low platelets (HELLP) syndrome, acute fatty liver of pregnancy and viral hepatitis such as hepatitis B and C. Given our patient’s Glasgow Alcoholic Hepatitis Score of 5 (on a scale of 5–12, with scores above 9 indicating a poor prognosis), the alcoholic hepatitis was classified as mild. Nevertheless, it likely contributed to the elevated PVP levels.

Our patient presented with symptomatic hypokalaemia, and the urinary potassium‐to‐creatinine ratio (uK/Cr) was consistent with renal potassium wasting. Potassium homoeostasis is regulated in the cortical collecting duct (CCD) of the nephron, where excretion is determined by two key factors: the potassium concentration within the CCD and the tubular flow rate. Potassium secretion from principal cells is driven by a lumen‐negative transepithelial potential. This negative charge is largely generated by aldosterone, which increases sodium reabsorption via activation of apical epithelial sodium channels (ENaC), creating a gradient favouring potassium secretion. In our case, however, the plasma aldosterone concentration was low, and urine electrolytes demonstrated low distal sodium and chloride delivery, suggesting that enhanced lumen negativity was not the primary mechanism of potassium loss. Instead, the renal potassium wasting was better explained by an increased tubular flow rate in the CCD, likely secondary to water diuresis. Furthermore, as the patient’s polyuria persisted despite correction of the hypokalaemia, hypokalaemia‐induced nephrogenic DI was considered unlikely.

The water deprivation test is discouraged in pregnancy due to the risks of maternal dehydration and potential adverse maternal and foetal outcomes [1, 12]. To demonstrate the presence of PVPs, we administered exogenous AVP, which elicited no renal response in terms of urinary concentration or reduction in urine volume. In contrast, administration of dDAVP resulted in an immediate renal response. This is because the cleavage site for PVPs on dDAVP differs from that of AVP; thus, the clearance of dDAVP is not affected by the presence of PVPs. However, there are concerns regarding the administration of AVP during pregnancy. AVP and oxytocin (OT) are posterior pituitary nonapeptides, and both hormones share a similar structure with just two amino acid differences in their sequence. It has been demonstrated that AVP interacts with OT receptors with a comparable affinity in animal models [13]. Therefore, there are concerns that the administration of AVP during pregnancy may induce uterine contractions. The precise role of AVP in myometrial signalling and parturition remains incompletely understood [14–16]. To confirm the presence of vasopressinase, AVP was administered. We were cognisant of the theoretical risk of premature labour associated with AVP administration; therefore, we counselled our patient prior to performing the test, and informed consent was obtained. She was admitted to a high‐care setting, and cardiotocography (CTG) was performed during the procedure to monitor foetal well‐being and uterine activity. The dosages of AVP and dDAVP used during the diagnostic tests were based on previous case series [17–19].

An alternative test is the measurement of serum AVP levels; however, these assays have significant limitations and complexity, such as a short half‐life, low molecular stability and high binding to platelets [20, 21]. Where the interpretation of these tests is complicated or inconclusive, copeptin testing must be considered, with hypertonic saline–stimulated copeptin shown to be superior to water deprivation–stimulated copeptin [22]; however, our laboratory could not perform this test. A key limitation of this case was the lack of confirmatory biochemical and placental analyses. Although the placenta was submitted for evaluation of vasopressinase synthesis, this could not be performed by our pathology laboratory, as it is not a routine test at our institution. In addition, copeptin measurement was also unavailable at our centre.

Lymphocytic hypophysitis is a rare autoimmune disorder characterised by lymphocytic infiltration and is categorised into lymphocytic adenohypophysitis and LINH. The latter leads to destruction and fibrosis of the pituitary infundibulum and pars nervosa, occurring more frequently in men [23]. In contrast to lymphocytic adenohypophysitis—which predominantly affects women, particularly in the peripartum period [23]—patients typically present with features of DI; however, the synthesis of OT and AVP remains intact, as these hormones are produced in the hypothalamic nuclei. Rather, the defect lies in impaired transport along the neurohypophyseal pathway [23]. We identified a single case describing both PVP‐DI and LINH as contributing mechanisms for DI during pregnancy [5]. The case involved a 39‐year‐old woman who presented at 31 weeks’ gestation with polyuria and polydipsia. Antepartum plasma AVP levels were inappropriately low, while vasopressinase levels exceeded 1000 times the upper limit of normal. She underwent a caesarean section due to the development of acute kidney injury. DDAVP was initiated immediately postpartum, resulting in rapid symptom resolution, and was discontinued by day 30 postpartum without recurrence of symptoms; however, at 1 month postpartum, a hypertonic saline infusion test and the presence of anti–rabphilin‐3A antibodies—highly specific for LINH—along with repeat MRI demonstrating a partial reappearance of the posterior pituitary ‘bright spot’, confirmed the diagnosis of LINH. This case closely parallels ours, particularly with respect to the discontinuation of dDAVP and the delayed diagnosis of LINH.

In conclusion, the accurate diagnosis and management of DI depend on a thorough understanding of normal renal physiology and the pathophysiological mechanisms specific to pregnancy. Clinicians should maintain a high index of suspicion for vasopressinase‐induced DI in pregnant patients presenting with hypokalaemia, polyuria due to water diuresis and hepatic dysfunction of any aetiology. Importantly, central causes of DI should not be overlooked; continued vigilance is warranted during the postpartum period, even when initial investigations suggest vasopressinase as the underlying mechanism.

## Author Contributions

Writing–original draft: Philani Ezekiel Mkhize; writing–review and editing: Philani Ezekiel Mkhize, Pauli van Heerden, Tholakele Sabela, Mogamat Razeen Davids and Mogamat‐Yazied Chothia; conceptualisation: Mogamat‐Yazied Chothia.

## Funding

The authors received no financial support for the research, authorship and/or publication of this article.

## Consent

The patient gave written informed consent, and the Health Research Ethics Committee (HREC) of Stellenbosch University granted permission to publish this case report (HREC reference number: C25/06/020; Project identification: 33625).

## Conflicts of Interest

The authors declare no conflicts of interest.

## Data Availability

The data that support the findings of this study are available on request from the corresponding author. The data are not publicly available due to privacy or ethical restrictions.
